# Twenty common errors in the prevention, diagnosis, and treatment of fracture-related infection (FRI)

**DOI:** 10.5194/jbji-11-219-2026

**Published:** 2026-04-13

**Authors:** Goran Georgievski, Nike Walter, Ronald Man Yeung Wong, Irene Katharina Sigmund, Ashok Kanuri, Christian Heiss, Markus Rupp

**Affiliations:** 1 Department of Trauma, Hand and Reconstructive Surgery, University Hospital Giessen, Justus Liebig University of Giessen, Giessen, Germany; 2 Helmholtz Centre Munich, German Research Center for Environmental Health, Institute of Virology, Munich, Germany; 3 Department of Orthopaedics & Traumatology, The Chinese University of Hong Kong, Hong Kong, China; 4 Department of Orthopaedics and Trauma Surgery, Medical University of Vienna, Vienna, Austria; 5 Department of Orthopedics and Traumatology, Biruni University, Istanbul, Türkiye

## Abstract

Fracture-related infections are among the most serious complications following osteosynthesis. They jeopardize fracture healing, prolong treatment duration, and can lead to loss of function or even amputation. Despite established standards, avoidable errors continue to occur in clinical practice. Fracture-related infections not only compromise healing but also significantly reduce life expectancy, and increase morbidity and mortality. The standardization of procedures is essential to improve outcomes and ensure consistent high-quality care. This article describes 20 common errors in the prevention, diagnosis, and treatment of fracture-related infection. For each error, the clinical consequences and practical recommendations are provided. The aim is to improve treatment quality and patient safety by identifying and avoiding typical decision-making pitfalls. Consistent standardization of surgical and microbiological procedures, interdisciplinary collaboration, and structured follow-up care are essential prerequisites for successful infection management.

## 1 Introduction

Fracture-related infections (FRIs) are among the most severe complications following osteosynthesis and remain a relevant clinical problem despite increasingly standardized treatment pathways (Walter et al., 2021, 2024). In the areas of prevention, diagnostics, and treatments, significant gaps persist between recommended and clinical practice. The current management of FRIs across Europe is highly variable, often lacking standardized treatment protocols.

In prevention, established measures such as correct perioperative antibiotic prophylaxis, consistent optimization of relevant risk factors, or structured soft-tissue management are often not fully applied (Bezstarosti et al., 2019; Metsemakers et al., 2020). Operative workflows, timing decisions, and hygiene standards are repeatedly compromised by organizational constraints or misjudgments. It would be helpful to explore why evidence-based practices are not more widely adopted, considering the psychological and emotional barriers that influence clinical decision-making. Addressing these issues could improve consistency and outcomes in FRI treatment.

Diagnostic errors arise from insufficiently structured clinical assessment, inconsistent use of laboratory and imaging modalities, and inadequate standardization of microbiological sampling (Rupp et al., 2024). Missing or misinterpreted diagnostic steps frequently lead to infections being overlooked, detected too late, or incorrectly classified – despite clearly defined criteria and diagnostic algorithms (McNally et al., 2020; Govaert et al., 2020).

In treatments, unclear indications, inadequate surgical strategies, and non-standardized antimicrobial concepts continue to negatively influence outcomes (Sigmund et al., 2025). Frequent issues include incomplete implant removal, insufficient debridement, delayed soft-tissue coverage, and inappropriate antibiotic regimens. Despite the availability of recommendations and increasing literature on FRIs, these errors are repeatedly observed in clinical practice (Isler et al., 2025; Perdomo-Lizárraga et al., 2025).

With this background, there is a considerable need to identify typical decision-making errors and focus more on the prevention as well on standardized methods to improve patient safety and treatment outcomes in FRIs.

## 2 Typical errors in the prevention of fracture-related infection

### 2.1 Error number 1: missing or delayed perioperative antibiotic prophylaxis in closed fractures

A common error in the operative management of closed fractures is omitting or incorrectly timing the administration of a first-generation cephalosporin. Missing prophylaxis or administering it outside the 0–60 min window before incision results in inadequate tissue concentrations with bacterial contamination, which demonstrably increases the risk of FRI (De Jonge et al., 2017; Ali et al., 2025; Bratzler et al., 2013).

#### Recommended approach

A single preoperative dose of 2 g cefazolin should be administered 0–60 min before skin incision. In obese patients ( ≥100 –120 kg), the dose must be increased to 3 g (Abele-Horn et al., 2024). Due to its short half-life and the extensive surgical procedures, re-dosing is necessary after 3 h or with blood loss > 1.5 L (Abele-Horn et al., 2024). This standardized prophylaxis significantly lowers the risk of FRI and represents the evidence-based standard for closed long-bone fractures (Table 1).

**Table 1:** Standardized perioperative antibiotic prophylaxis for closed fractures.

Aspect	Recommendation
Antibiotic	Cefazolin
Standard dose	2 g IV
Timing of administration	15–60 min before skin incision
Dose adjustment for obesity	3 g IV for patients ≥ 100–120 kg
Re-dosing	After 3 h of surgery or with blood loss > 1.5 L
Rationale	Short half-life of cefazolin
Evidence	Significantly reduces the risk of fracture-related infection; evidence-based standard for all closed fractures.

djusted from the recommendation from Perioperative and Periinterventional Antibiotic Prophylaxis in Surgery (Bratzler et al., 2013).

### 2.2 Error number 2: failure to use local antibiotics despite clear indications in open fractures

A frequent mistake in open contaminated fractures is relying solely on systemic antibiotics and omitting local antibiotic carriers, despite their potential to additionally reduce infection risk. In cases of substantial contamination, segmental bone loss, or compromised soft tissues, the infection risk remains elevated even with correct systemic prophylaxis.

#### Recommended approach

The therapeutic standard consists of thorough surgical debridement, generous irrigation, stable fixation, and immediate systemic antibiotic administration. Local antibiotics (e.g., beads, powder, absorbable carriers) should be considered whenever possible. Meta-analyses demonstrate a notable reduction in infection rates (4.6 % vs. 16.5 %), although evidence remains heterogeneous (Awad et al., 2023). Given their low rate of adverse effects, local antibiotics are advisable as an adjunct to support standard therapy and reduce FRI risk.

### 2.3 Error number 3: inadequate, delayed, or unnecessarily prolonged systemic antibiotic therapy in open fractures

Systemic antibiotics are often administered too late, in insufficient doses, or for far longer than necessary. Delayed administration prevents the timely achievement of effective tissue concentrations during maximal contamination. Conversely, treatment durations exceeding 24 h in Gustilo I–II or 72 h in Gustilo III fractures do not reduce FRI risk but increase the likelihood of side effects and resistance.

#### Recommended approach

Systemic antibiotics should be administered immediately along with debridement, irrigation, and stabilization. For Gustilo I–II injuries, 24 h prophylaxis with a first-generation cephalosporin (cefazolin) is recommended; alternatives exist primarily for β -lactam allergies (e.g., clindamycin) (Miller et al., 2011). For Gustilo III fractures, prophylaxis should not exceed 72 h or should end 24 h after soft-tissue closure, using an antibiotic with gram-negative and anaerobic coverage, such as piperacillin/tazobactam (Carver et al., 2017; Griffin et al., 2025) (Table 2). Gentamicin and metronidazole are no longer standard and should be used only for specific indications. Timely severity-adjusted prophylaxis remains central for infection prevention.

**Table 2:** Antibiotic management of open fractures, adjusted from Awad et al. (2023), Miller et al. (2011), Carver et al. (2017), and Lloyd et al. (2017).

Aspect	Recommendation
Initial administration	Immediate with debridement, irrigation, and stabilization
Antibiotic for Gustilo I–II	Cefazolin
Antibiotic for Gustilo III	Piperacillin/Tazobactam
Duration for Gustilo I–II	24 h
Duration for Gustilo III	72 or 24 h after soft-tissue closure
Alternatives for ß-Lactam allergy	Clindamycin for ß-lactam allergy and broad-spectrum antibiotics by Gustilo III (e.g.,
	aminoglycoside like gentamicin or fluoroquinolone)

### 2.4 Error number 4: insufficient preoperative risk assessment in patients with elevated infection risk

A major error is failing to systematically identify or optimize individual risk factors for FRI. Poorly controlled diabetes, ongoing smoking, peripheral arterial disease, obesity, malnutrition, or immunosuppressive therapies often remain unaddressed, despite their known impact on wound complications, delayed healing, and postoperative infections. All these factors should be reduced or optimized to minimize the risk from trauma to surgery, if applicable.

#### Recommended approach

Careful preoperative risk evaluation with documented screening for metabolic, vascular, immunological, and nutritional factors is required. The optimization of glucose control (perioperative target < 180 mg dL^−1^), smoking cessation, ensuring adequate nutrition, the adjustment of immunosuppressive medication, and the assessment of limb perfusion should occur before osteosynthesis or reconstruction (Roth et al., 2021; Lehmann, 2025). Consistent risk stratification and optimization significantly reduce complications and are essential components of modern FRI prevention.

### 2.5 Error number 5: missing or insufficient vascular assessment in compromised limbs

A clinically significant error is failing to identify a potentially ischemic or borderline-perfused limb in time. Subtle indicators such as prolonged capillary refill, diminished pulses, or atypical pain patterns are often overlooked. Inadequate perfusion leads to soft-tissue insufficiency, delayed healing, and markedly elevated FRI risk – especially in open fractures, high-energy trauma, vascular injuries, and in patients with peripheral artery diseases or diabetes.

#### Recommended approach

A structured vascular assessment should be performed early. Clinical examination should be supplemented with Doppler ultrasound and ankle brachial index (ABI). If perfusion is unclear, pulses are absent, or soft-tissue destruction is significant, CT angiography should be performed immediately (Aboyans et al., 2018; Romagnoli et al., 2021; Joseph et al., 2021). This applies not only in acute trauma but also in established FRI to evaluate the need for re-vascularization. Early vascular assessment improves soft-tissue conditions and reduces infection-related complications.

### 2.6 Error number 6: insufficient debridement and delayed soft-tissue coverage in open fractures

A major error is performing debridement too conservatively or too late. Remaining necrotic or contaminated tissue promotes bacterial persistence and biofilm formation, and significantly increases FRI risk. Delays in soft-tissue reconstruction further compromise tissue viability and promote infection (Kuripla et al., 2021; Cao et al., 2022).

#### Recommended approach

A thorough, complete debridement with removal of all devitalized tissue, extensive irrigation, and timely stabilization is required (Kuripla et al., 2021). Definitive soft-tissue coverage should occur within 72 h whenever possible (Cao et al., 2022). In complex defects or poor soft-tissue conditions, plastic surgery should be involved early to design an appropriate flap or reconstruction strategy (Powell-Bowns and Keating, 2024). Early interdisciplinary soft-tissue management reduces infection risk and supports successful bone healing.

## 3 Typical errors in the diagnosis of FRI

### 3.1 Error number 7: fistula formation or purulent drainage not recognized as definitive signs of infection

**Figure 1:**
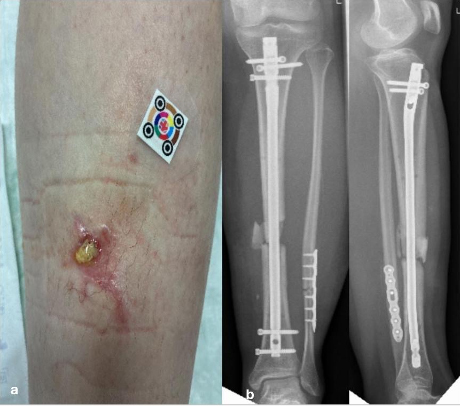
**(a)** A 16-year-old patient with a lower leg fracture classified as Gustillo–Anderson grade III. Initial management involved external fixation, followed by definitive fixation with intramedullary nailing. The patient is currently presenting with a fistula and a fracture-related infection (FRI), often misinterpreted as wound healing disturbance. **(b)** Anteroposterior and coronal radiographs demonstrating a lower leg fracture stabilized with intramedullary nailing. The images reveal a non-progressive fracture healing pattern with radiological features consistent with fracture-related infection.

Fistulas, purulent discharge, or persistent wound drainage are sometimes misinterpreted as postoperative irritation. Such misjudgments delay necessary diagnostic and surgical actions, allowing infection progression, jeopardizing implant stability, and risking systemic complications (Metsemakers et al., 2018). International FRI criteria define fistula, pus, or visible purulent drainage as definitive signs of infection (Fig. 1a).

#### Recommended approach

The presence of a fistula or purulent discharge indicates a manifestation of FRI and requires timely surgical intervention, including careful debridement, multiple deep-tissue samples, the restoration of stability, and targeted systemic and/or local antimicrobial therapy. Rapid structured management is crucial to prevent progression, chronic infection, and functional loss.

### 3.2 Error number 8: fistulography and fistula/wound swabs considered diagnostically relevant

Contrast fistulography is often misinterpreted as diagnostically meaningful. The visualized fistula tract does not necessarily reflect the true anatomical or pathological extent of infection. Similarly, microorganisms obtained from fistula or wound swabs are frequently mistaken for true infecting organisms, even though such tracts are almost always contaminated by skin or environmental flora (Bowler et al., 2001; Walker et al., 2020). Both practices can lead to false interpretations and incorrect treatment decisions.

#### Recommended approach

Microbiological diagnostics should rely exclusively on multiple deep intraoperative tissue samples taken from different locations. These specimens provide reliable pathogen identification and prevent mismanagement caused by contaminated superficial samples (Walker et al., 2020). Fistulography is not required to treat FRI successfully.

### 3.3 Error number 9: superficial or insufficient microbiological sampling

Diagnostic errors frequently arise from taking samples from superficial or irrelevant sites. Such samples are almost inevitably contaminated and lack diagnostic value. Taking fewer than three deep samples reduces sensitivity and increases the risk of missing relevant pathogens (Dudareva et al., 2021). Conversely, obtaining more than five samples increases cost and raises the probability that contaminants appear in at least two cultures and are mistakenly classified as relevant pathogens.

#### Recommended approach

A total of three to five deep-tissue samples, each obtained using separate instruments, should be collected. Superficial swabs must be avoided (Sousa et al., 2021). When possible, implant or screw sonication provides additional pathogen information and increases diagnostic accuracy (Bellova et al., 2021).

### 3.4 Error number 10: antibiotic administration before sampling

Antibiotics administered before collecting microbiological samples reduce the bacterial load and often result in false-negative cultures, complicating later therapeutic decisions and the interpretation of susceptibility results.

#### Recommended approach

All deep-tissue samples should be taken before antibiotic therapy begins. If clinically feasible, a two-week antibiotic-free interval before planned sampling increases diagnostic sensitivity (Hellebrekers et al., 2019; Loiez et al., 2025). In septic or life-threatening situations, patient safety has priority and immediate antibiotic therapy should be administered. In non-septic patients, tissue samples must be obtained before the first antibiotic dose, followed by prompt surgical and antibiotic treatment.

### 3.5 Error number 11: omission of histopathology

Deep-tissue samples are sometimes not sent for histological analysis, leading to loss of valuable diagnostic information. Histopathology can determine the inflammation grade, bone vitality, or presence of sequestra – findings that microbiology alone may not reveal, especially in low-grade infections (Glaudemans et al., 2019).

#### Recommended approach

All samples should undergo histological evaluation. Histopathology provides complementary insights that increase diagnostic reliability, particularly in subtle or chronic infections.

### 3.6 Error number 12: lack of cross-sectional imaging (CT/MRI) to determine infection extent

Relying solely on conventional radiographs often underestimates the true extent of infection. Radiographs show late changes and poorly visualize soft tissues, and may not detect osteomyelitis, sequestra, or implant loosening.

#### Recommended approach

CT should be used to assess bony structures, implant position, and sequestra. MRI is ideal for evaluating soft tissues, marrow involvement, and osteomyelitis extent (Foti et al., 2023; Alaia et al., 2021). When uncertainty persists, nuclear medicine imaging such as leukocyte scintigraphy or hybrid techniques can provide additional diagnostic clarity. In particular, PET CT scan is a useful tool that can provide information if hematogenous spreading of the infection is suspected.

## 4 Errors in the treatment of FRI

### 4.1 Error number 13: failure to systematically assess systemic signs of infection

Systemic manifestations of infection in the setting of FRI are frequently underestimated. Early indicators of sepsis – such as fever, tachycardia, hypotension, or altered mental status – may be missed, especially when local symptoms dominate clinical attention (Ludwick et al., 2022; Lourtet-Hascoët et al., 2025). In addition, essential laboratory tests are sometimes not obtained in a timely manner, and blood cultures are often omitted before antibiotics are initiated. This results in the delayed recognition of systemic infection, the loss of microbiological diagnostic yield, and the impaired ability to guide targeted therapy. Such omissions can allow progression to severe sepsis or septic shock, significantly worsening patient outcomes.

#### Recommended approach

Whenever systemic involvement is suspected, two sets of blood cultures must be drawn before starting antibiotics, ensuring optimal pathogen recovery. In parallel, CRP and leukocyte count should be measured, and procalcitonin can be added when severe infection or sepsis is a concern (Chomba et al., 2020). A structured repeated assessment of vital signs and inflammatory markers is essential for the early identification of deterioration and the timely escalation of care.

### 4.2 Error number 14: inadequate implant strategy despite confirmed infection

Implants are sometimes kept in place even when mechanical instability or long-standing infection is evident, two situations in which successful biofilm eradication is virtually impossible. The retention of a loose or chronically infected implant allows persistent microbial reservoirs to remain and undermines both infection control and fracture healing. Equally problematic is premature re-osteosynthesis without adequate debridement or hesitancy to perform a technically feasible one-stage exchange. These decisions often lead to repeated surgeries, prolonged infection, and impaired functional outcomes (Jones et al., 2023). It emphasizes that implant stability and the ability to achieve radical debridement are decisive factors for selecting the correct surgical strategy.

#### Recommended approach

A one-stage exchange is appropriate when the soft tissues are intact, a stable biological environment exists, and complete debridement can be performed without major bone loss. If local conditions are unfavorable – e.g., compromised soft tissue, segmental defects, or uncertain debridement – a staged procedure with temporary external fixation or an antibiotic spacer is recommended. Implant retention can be acceptable only in early infections with a demonstrably stable construct (Baertl et al., 2024; Alt et al., 2024).

### 4.3 Error number 15: retention of an intramedullary nail despite manifest infection

Leaving an infected intramedullary nail in situ allows a persistent biofilm reservoir to remain within the medullary canal. Because the canal offers a protected, poorly penetrated niche, pathogens can survive despite systemic antibiotics and repeated superficial procedures. This frequently leads to treatment failure, recurrent infection, delayed fracture healing, and the need for multiple revision surgeries. An infected intramedullary implant cannot be reliably sterilized in vivo; therefore, simple irrigation or limited debridement without removing the implant is inadequate (Baertl et al., 2024; Alt et al., 2024; Neyt et al., 2024). Retaining the nail maintains mechanical stability but sacrifices infection control, ultimately prolonging morbidity and undermining the entire treatment strategy.

#### Recommended approach

Management requires complete removal or exchange of the infected nail. For complete removal, following extraction, the medullary canal must be thoroughly debrided and reamed to eliminate infected tissue and biofilm (Baertl et al., 2024; Alt et al., 2024). Temporary stabilization – typically via external fixation or an antimicrobial-coated intramedullary spacer – can safely bridge the interval until definitive reconstruction or re-osteosynthesis is feasible. This staged approach provides both stability and reliable infection eradication.

### 4.4 Error number 16: limited “local revisions” or isolated fistula excision

Small superficial procedures – such as minor wound revisions, abscess drainage, or isolated fistula excision – do not reach the true infectious focus in FRI. These limited interventions fail to address biofilm-bearing tissue and contaminated structures, and therefore cannot achieve durable infection control. The underlying infection persists, often unnoticed, and almost inevitably leads to recurrence, repeated surgeries, implant loosening, chronic osteomyelitis, or prolonged treatment courses. This misconception remains a common reason for therapeutic failure, delayed healing, and unnecessary morbidity.

#### Recommended approach

Successful management requires a radical, complete debridement down to vital, bleeding tissue. All necrotic, contaminated, or potentially biofilm-containing structures – including infected implant components, sequestra, fibrotic tissue, and nonviable soft tissue – must be removed to achieve definitive infection control. The surgical strategy must aim for comprehensive eradication of the infectious source, not for superficial correction (Baertl et al., 2024; Neyt et al., 2024). Only a thorough and systematic debridement ensures sustainable infection clearance.

### 4.5 Error number 17: incomplete debridement and continuation of unstable osteosynthesis

Debridement is sometimes performed too cautiously due to concern about destabilization, or unstable fixation is maintained despite infection. Both promote chronic infection and inhibit fracture healing.

#### Recommended approach

Debridement must be complete. A living wound means well-vascularized soft and bony tissue has to be achieved. This is essential to enable infection eradication and later healing of the fracture and bone defect (Baertl et al., 2024). Fixation may only be retained if demonstrably stable, otherwise exchange or temporary fixation is necessary to ensure infection control and bone healing.

### 4.6 Error number 18: incorrect or excessively prolonged antibiotic therapy

Antibiotic therapy in FRI is often initiated too broadly or continued for too long without alignment to guideline-based empirical principles (Metsemakers et al., 2018). Empiric treatment is sometimes started before adequate sampling or maintained unchanged after culture results. Excessively broad regimens, including unnecessary MRSA coverage or carbapenems, are frequently used despite low local MRSA prevalence and the predominance of susceptible staphylococci and streptococci. Culture-guided de-escalation and early oral transition are often delayed, leading to overtreatment, avoidable toxicity, and increased resistance pressure.

#### Recommended approach

Empiric therapy should begin after sampling and follow the guideline draft, which recommends aminopenicillin/ β -lactamase inhibitors or second/third generation cephalosporins as standard monotherapy, reflecting the typical pathogen spectrum. Routine MRSA coverage is unnecessary and should be reserved for defined risk factors; very broad combinations should be limited to severe contamination, critical illness, or high MDR (Multidrug-Resistant) suspicion. Therapy must be promptly narrowed once cultures are available. An early switch to high-bioavailability oral agents is encouraged, supported by the OVIVA (Oral Versus IntraVenous Antibiotics) trial showing non-inferiority to prolonged IV therapy. Total duration should follow implant status: ∼6 weeks without implants and 6 through 12 weeks when implants are retained or re-implanted, according to stability and debridement quality (Rupp et al., 2024; Asim et al., 2024; Bernard et al., 2021).

### 4.7 Error number 19: delayed soft-tissue coverage and prolonged VAC therapy

In many cases, vacuum-assisted closure (VAC) therapy is continued far beyond its intended purpose as a short-term bridging measure. Clinicians may wait for repeated dressing changes, subjective wound improvement, or even a “negative swab” before planning definitive soft-tissue reconstruction. However, swab cultures have no diagnostic or prognostic value in this context; and prolonged VAC use can desiccate tissues, increase bacterial burden, and delay the necessary reconstruction (Boone et al., 2010). Each additional day without stable soft-tissue coverage increases the risk of persistent infection, implant exposure, and compromised fracture healing. These delays represent a significant, yet preventable, contributor to treatment failure in FRI.

#### Recommended approach

Soft-tissue coverage should be achieved early, ideally within 72 h after a thorough radical debridement (Steiert et al., 2009). VAC therapy should be used only as a short-term (24–72 h) temporary measure to bridge until definitive coverage is available. Operative timing must be guided by surgical readiness and tissue viability – not by superficial swabs or repeated VAC cycles (Cao et al., 2022; Pincus et al., 2019; Godina, 1986).

### 4.8 Error number 20: lack of a multidisciplinary approach

Treatment decisions are often made without involving infectious disease specialists, microbiologists, radiologists, or plastic surgeons. This lack of coordination compromises sampling, debridement strategy, implant decisions, and soft-tissue management.

#### Recommended approach

A multidisciplinary team (MDT) should be involved from the first suspicion of FRI. Trauma surgery, infectious diseases, microbiology, radiology, and plastic surgery should collaborate, with additional specialists included depending on patient comorbidities (Metsemakers et al., 2018; Hanssen et al., 2025). Coordinated planning improves diagnostic quality, surgical strategy, and therapeutic success.

## 5 Conclusion

Effective FRI management requires a structured phase-oriented strategy. Prevention begins with patient optimization. Diagnostic success relies on standardized sampling and appropriate imaging. Therapeutically, radical debridement, stable fixation, adequate soft-tissue coverage, and targeted antimicrobial therapy are essential. Many complications can be avoided through interdisciplinary collaboration and consistent follow-up.

## Data Availability

The data and information used in this research are based on existing clinical guidelines, published studies, and expert consensus on fracture-related infections. All references and recommendations are drawn from publicly available medical literature, clinical protocols, and established standards in the field. No new raw data or proprietary datasets were generated for this study. For further details or access to specific sources, please refer to the original publications and guidelines cited in the research.
